# Cytotoxicity of InP/ZnS Quantum Dots With Different Surface Functional Groups Toward Two Lung-Derived Cell Lines

**DOI:** 10.3389/fphar.2018.00763

**Published:** 2018-07-13

**Authors:** Ting Chen, Li Li, Gaixia Xu, Xiaomei Wang, Jie Wang, Yajing Chen, Wenxiao Jiang, Zhiwen Yang, Guimiao Lin

**Affiliations:** ^1^Department of Physiology, School of Basic Medical Sciences, Shenzhen University Health Sciences Center, Shenzhen, China; ^2^Key Laboratory of Optoelectronics Devices and Systems of Ministry of Education, College of Optoelectronic Engineering, Shenzhen University, Shenzhen, China

**Keywords:** InP/ZnS quantum dots, cytotoxicity, cellular uptake, cell apoptosis, ROS generation

## Abstract

Although InP/ZnS quantum dots (QDs) have emerged as a presumably less hazardous alternative to cadmium-based QDs, their toxicity has not been fully understood. In this work, we report the cytotoxicity of InP/ZnS QDs with different surface groups (NH_2_, COOH, OH) toward two lung-derived cell lines. The diameter and the spectra of InP/ZnS QDs were characterized and the hydrodynamic size of QDs in aqueous solution was compared. The confocal laser scanning microscopy was applied to visualize the labeling of QDs for human lung cancer cell HCC-15 and Alveolar type II epithelial cell RLE-6TN. The flow cytometry was used to confirm qualitatively the uptake efficiency of QDs, the cell apoptosis and ROS generation, respectively. The results showed that in deionized water, InP/ZnS-OH QDs were easier to aggregate, and the hydrodynamic size was much greater than the other InP/ZnS QDs. All these InP/ZnS QDs were able to enter the cells, with higher uptake efficiency for InP/ZnS-COOH and InP/ZnS-NH_2_ at low concentration. High doses of InP/ZnS QDs caused the cell viability to decrease, and InP/ZnS-COOH QDs and InP/ZnS-NH_2_ QDs appeared to be more toxic than InP/ZnS-OH QDs. In addition, all these InP/ZnS QDs promoted cell apoptosis and intracellular ROS generation after co-cultured with cells. These results suggested that appropriate concentration and surface functional groups should be optimized when InP/ZnS QDs are utilized for biological imaging and therapeutic purpose in the future.

## Introduction

QuantumDot (QD), also known as semiconductor nanometer microcrystal, is currently one of the most popular nano materials (Pohanka, 2017) owing to its unique optical features, such as wide absorption spectrum, narrow emission spectrum, long fluorescence lifetime, high intensity and strong resistance to photobleaching, etc. (Bruchez et al., 1998). QDs has been developed rapidly and widely used in biomedical research such as biological sensing, imaging, food quality control and biochip technology (Lin et al., 2015b; Peng et al., 2018; Zhou et al., 2018). However, more QDs nanoparticles are entering into the environment with the increasing development of QDs, more QD exposure hazards from product industries and research institutions resulting in toxicity *in vitro* and *in vivo* are increased. Thus, serious concerns have been raised about the biosafety of QDs due to limited understanding of the toxicological behavior of quantum dots (QDs).

The impacts of QDs on the environment and human being have been put forward recently by many scientists and organizations (Manshian et al., 2016, 2017; Liu et al., 2017). The toxicity of QDs has been evaluated using multiple *in vitro* cell models including human bronchial epithelial cells (Zheng et al., 2018), HepG2 cell line (Paesano et al., 2016), macrophages and lymphocytes (Wang X. et al., 2016) and *in vivo* animal models such as mice (Liu et al., 2017), rat (Ma-Hock et al., 2012), and non-human primate (Ye et al., 2012). So far, the collected data is still inconclusive because many factors are responsible for the toxicity of QDs. QD-induced toxicity is closely related to their surface properties (including shell, ligand and surface modifications), size, biological model, and exposure route and time (Oh et al., 2016).

Although some progress has been made in the toxicity study on QDs, most of the research still focused on cadmium-containing QDs, such as CdTe, CdS, and CdSe. Studies have shown that the release of cadmium ion from cadmium-containing QDs caused damage to cells (Li et al., 2009) or organs (Wang M. et al., 2016). The risk of cadmium exposure and the toxicity of cadmium-containing QDs (Mo et al., 2017) have initiated a heated debate over whether or not to keep on pursuing the translation of QDs into clinical research and applications.

In order to overcome this problem, several strategies have been proposed, such as the generation of cadmium-free QD dots. InP/ZnS (indium phosphide/zinc sulfide) nanocrystals are the most commonly used core/shell cadmium-free QDs. InP/ZnS QDs have appeared to be a less hazardous nanocrystal in comparison with cadmium-containing nanoparticles (Brunetti et al., 2013) since they are free of cadmium and also have greater degree of covalent bonding, comparing to those made up of group II–VI elements. Chibli et al found a small amount of hydroxyl radical formed under visible illumination of biocompatible InP/ZnS QDs, comparable to what is seen with CdTe, indicating that InP/ZnS QDs are a useful alternative to cadmium-containing QDs (Chibli et al., 2011). Previously, we have systematically studied the *in vivo* biodistribution and long term toxicity of InP/ZnS QDs in BALB/c mice (Lin et al., 2015a). We found that accumulation of indium element from injected InP/ZnS QDs still remained at major organs even after 84 days of injection. But hematology, blood biochemistry, and histological analysis indicated that there are no acute toxic effects.

Although InP/ZnS QDs have emerged as a presumably less hazardous alternative to cadmium-based particles, their toxicity has not been fully observed. In comparison to cadmium-containing QDs, the understanding of InP QD toxicity is still in its infancy stage, and little is known about their toxicological effects (Soenen et al., 2014). Lung is the first exposed target for inhaled nanoparticles, and it also receives the entire cardiac output, which makes the risk of lung injury high. Previously, Ho et al reported that pulmonary exposure to cadmium-based QDs will result in persistent inflammation and granuloma formation in the mouse lung (Ho et al., 2013). Furthermore, surface coating to influence the disposition and toxicity of QDs in animal lungs (Roberts et al., 2013). Injures of lung will seriously damage the respiratory function and cause serious lung disease. Study of cytotoxicity toward these two lung-derived cell lines help to understand the impact of InP/ZnS QDs on respiratory function.

In this study, we investigated the *in vitro* toxicity of InP/ZnS terminated with different surface groups (COOH, NH_2_ and OH respectively) on two lung-derived cell lines, human lung cancer cell HCC-15 and Alveolar epithelial type II (AEII) cell RLE-6TN, which are common cell models for studying respiratory toxicity of nanoparticles (Zienolddiny et al., 2000; Schwotzer et al., 2018). AEII cells are an important component of the respiratory defense system against foreign material, including nanoparticles. They are responsible for production and recycling of lung surfactant, play a role in turnover of the alveolar epithelia, and bear the ability to transform into alveolar epithelial type I (AEI) cells (e.g., for replacement of damaged cells) (Schwotzer et al., 2018). HCC-15 cells is immortalized cell lines derived from squamous cell lung cancer, which is the second most common type of lung cancer, usually originating in the large airways in the central part of the lungs (Wu et al., 2013). We found that all the QDs could enter the cells at similar percentage when the dose reached 20 μg/mL. While at the dose of 2 μg/mL, the uptake efficiency of QDs with hydroxyl was relatively lower than the others. All these QDs caused the cell proliferation inhibition, cell apoptosis and ROS generation. These results suggested that we should optimize the concentration of quantum dot within a safe range while using InP/ZnS QDs as optical probes for cell imaging or other clinical applications.

## Materials and Methods

### Preparation and Characterization of QDs

InP/ZnS QDs were purchased from Najingtech Company. The morphology images of InP/ZnS QDs dispersed in toluene were obtained with a transmission electron microscope (TEM) (Tecnai G2 F20 S-TWIN, FEI, United States) operating at an accelerating voltage of 200 kV at room temperature. When inversed from oil phase to aqueous phase, InP/ZnS QDs was coated with a polymer layer and terminated with carboxyl, hydroxyl and amino surface groups respectively. Before experiment, the content of indium element in the three QDs solutions was measured by ICP-MS (7500C1, Agilent, United States) analysis and the result was normalized to equalize the concentrations of the three QDs. The absorption spectra of InP/ZnS QDs were measured by a UV-Vis spectrophotometer (Cary 5000, Agilent, United States). The photoluminescence emission spectra were determined by a Fluorescence spectrophotometer (F-4600, Hitachi, Japan) with an excitation wavelength of 400 nm. The hydrodynamic size distribution of InP/ZnS QDs was obtained using a dynamic light scattering (DLS) machine (Zetasizer Nano ZS, Malvern, United Kingdom).

### Cell Culture

The human lung cancer cell HCC-15 and Alveolar type II epithelial cell RLE-6TN were obtained from American Type Culture Collection (ATCC) and cultured in Dulbecco’s Modified Eagle’s Medium (DMEM, Gibco, United States) supplemented with 10% fetal bovine serum (FBS, Gibco, United States) and 100 U penicillin/streptomycin (Gibco, United States). All cells were cultured at 37°C in humidified atmosphere with 5% CO_2_.

### Confocal Laser Scanning Microscopy

The day before imaging, cells were planted onto 35 mm confocal dishes (Thermo Scientific^TM^ Nunc^TM^, United States) to give 30–50% density. Cells were left untreated or co-incubated with 2 μg/mL InP/ZnS QDs. After 4–6 h of incubation, the culture medium was removed and cells were washed with phosphate-buffered saline (PBS) twice, fixed with 4% paraformaldehyde for 15 min. Then the paraformaldehyde solution was abandoned and the cell nucleuses were stained with DAPI for 5 min. The confocal images were obtained using a laser scanning confocal microscope (LSCM, TCS SP5, Leica, DEU).

### Uptake Efficiency Detected by Flow Cytometry

The day before experiment, cells were seeded into 6-well plates in medium to give 30–50% density. Cells were left untreated or co-incubated with InP/ZnS QDs (2 and 20 μg/mL, respectively). After 4–6 h of incubation, the culture medium was removed and cells were washed with PBS, harvested by trypsin (Gibco). After centrifugation, cells were resuspended in 300 μL PBS solutions and analyzed immediately by a flow cytometer (FACS Aria II, BD, United States).

### Cell Viability Detected by MTT Assay

The cell viability of HCC-15 and RLE-6TN cells were evaluated by MTT (Sigma-Aldrich, United States) assay. Cells were seeded in 96-well plates (5 × 10^3^ cells/well) and incubated with different concentrations of InP/ZnS QDs for 24 or 48 h. The MTT solution (5 mg/mL) was added into cells for 10 μL/well. After 4 h incubation, the culture medium was removed carefully and DMSO (150 μL/well) was added to sufficiently dissolve the blue crystals. The plates were gently shaken for 5 min and absorbance was measured with a microplate reader (Multiskan FC, Thermo Fisher, Finland) at a wavelength of 570 nm. The cell viability was calculated by normalizing the absorbance of the sample well against that of the control well and expressed as a percentage, assigning the cell viability of non-treated cells as 100%.

### Cell Apoptosis Detected by Flow Cytometry

The cell apoptosis was measured by Annexin V-FITC Apoptosis Detection Kit (BD Pharmingen^®^, United States). The day before assay, cells were planted onto 6-well plates. For apoptosis detection, cells were left untreated or exposed to InP/ZnS QDs for 24 or 48 h. All cells were dissociated with trypsin solution (Gibco, United States) without EDTA and collected by centrifugation at 1,500 rpm for 5 min. After washed with pre-cooling PBS, cells were resuspended in 300 μL 1 × binding buffer. Five microliter Annexin V-FITC solution was added into cells and cells were incubated for 15 min at room temperature and protected from light. The signals of FITC fluorescence were detected by a flow cytometer (FACSAria II, BD, United States).

### Detection of Reactive Oxygen Species (ROS)

The production of cellular ROS was detected by the carboxy-dichlorofluorescein diacetate (carboxy-DCFH-DA) assay kit (Sigma-Aldrich, United States). Cells were planted onto 6-well plates and left untreated or treated with InP/ZnS QDs. After 4–6 h of stimulation, the culture medium was removed and 10 μM DCFH-DA solutions was added into cells to load the probe. Cells were incubated for 20 min at 37°C with 5% CO_2_ and washed with serum-free medium twice to remove the extra DCFH-DA. Cells were collected and analyzed immediately by a flow cytometer (FACS Calibur, BD, United States).

### Statistical Analysis

All experimental data were expressed as mean ± standard deviation (SD). Multigroup comparisons of the means were carried out by one-way analysis of variance (ANOVA) test. Dunnett’s test was used to compare the differences between the experimental groups and the control group. All statistical calculations were performed with the SPSS 11.0 software package. The statistical significance for all tests was set at *p* < 0.05.

## Results

### Characterization of InP/ZnS QDs

**FIGURE 1:**
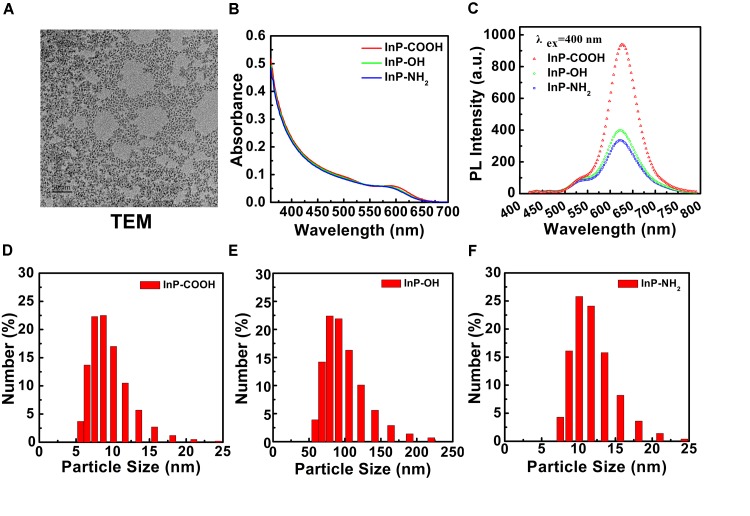
Characterization of InP/ZnS QDs with various surface functional groups. **(A)** TEM image of InP/ZnS QDs dispersed in toluene. Scale bar: 50 nm. **(B)** Absorption spectra and **(C)** photoluminescence (PL) spectra of InP/ZnS-COOH QDs, InP/ZnS-OH QDs, and InP/ZnS-NH_2_ QDs, respectively. Hydrodynamic size distributions of **(D)** InP/ZnS-COOH QDs, **(E)** InP/ZnS-OH QDs, and **(F)** InP/ZnS-NH_2_ QDs dispersed in deionized water.

The TEM image of InP/ZnS QDs before surface modifications was shown in **Figure 1A**. It demonstrated a relatively monodispersed size distribution with averaged size of ∼5 ± 0.5 nm. After coating with a polymer layer and terminated with carboxyl, hydroxyl or amino surface groups, we performed the FT-IR spectra analysis of the QDs which indicating successful functionalization of the three surface groups (Supplementary Figure S1). The InP/ZnS QDs modified with carboxyl, hydroxyl and amino groups, respectively, exhibited consistent absorption spectra with the same absorption peak around 580 nm (**Figure 1B**). Under excited by 400 nm light source, the three QDs all exhibited relatively symmetrical photoluminescence spectra with the emission peak around 625 nm (**Figure 1C**). The average hydrodynamic diameters of the aqueous QDs characterized by DLS technique were 9.267 ± 2.769 nm, 11.70 ± 3.031 nm and 97.79 ± 31.74 nm for InP/ZnS-COOH QDs, InP/ZnS-NH_2_ QDs and InP/ZnS-OH QDs, respectively (**Figures 1D–F**). Accordingly, the zeta potentials of these QDs were -43.1 ± 8.13 mV, -54.6 ± 7.06 mV, -40.5 ± 8.33 mV, respectively.

### Uptake of Quantum Dots by Lung-Derived Cells

**FIGURE 2:**
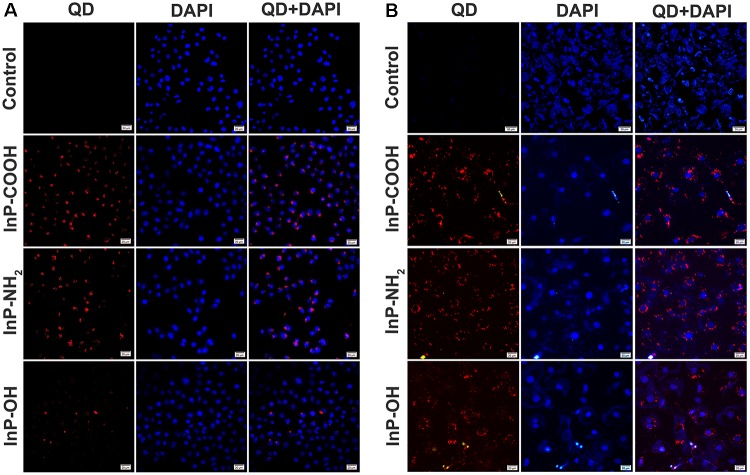
Fluorescence images of **(A)** HCC-15 and **(B)** RLE-6TN cells treated with (i) Blank, (ii) InP/ZnS-COOH, (iii) InP/ZnS-NH_2_, and (iv) InP/ZnS-OH QDs for 4 h. The cell nucleuses are stained with DAPI (in blue), and the signals from QDs are in red. Scale bar: 20 μm.

The confocal images of HCC-15 and RLE-6TN cells treated with the 2 μg/mL QDs for 4 h were shown in **Figure 2**. Compared with the control group, obvious red signals from QDs were observed around the cell nucleuses in the QDs treated groups, which indicated that the QDs could be taken up by human lung cancer cell HCC-15 and Alveolar type II epithelial cell RLE-6TN. It can also be clearly seen that the intake of InP/ZnS-OH QDs is relatively lower than InP/ZnS-COOH QDs and InP/ZnS-NH_2_ QDs.

**FIGURE 3:**
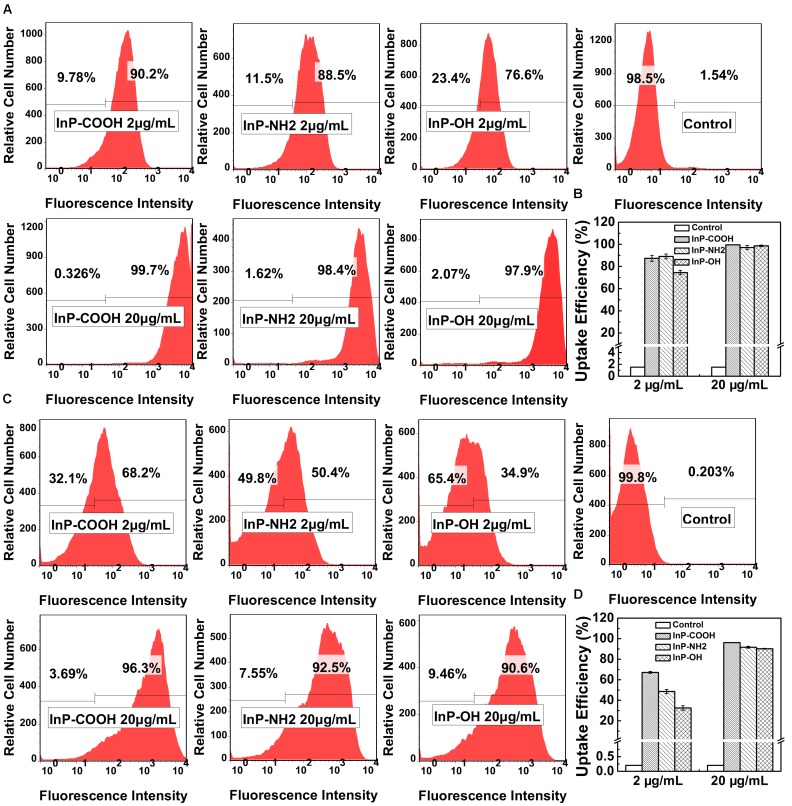
The uptake efficiency of InP/ZnS-COOH, InP/ZnS-NH_2_, and InP/ZnS-OH QDs by cells treated for 4 h. Representative plots of the fluorescence intensity in **(A)** HCC-15 and **(C)** RLE-6TN cells treated with 2 and 20 μg/mL QDs. The uptake efficiency of **(B)** HCC-15 and **(D)** RLE-6TN cells, evaluated from experiments shown in **(A,C)**. Values are means ± SD, *n* = 3.

To further quantitatively evaluate the uptake efficiency of QDs by HCC-15 and RLE-6TN cells, flow cytometry analysis was performed (**Figure 3**). For HCC-15 cells treated with 2 μg/mL QDs, the uptake efficiency of InP/ZnS-COOH, InP/ZnS-NH_2_ and InP/ZnS-OH are 87.4 ± 2.67%, 89.0 ± 2.15%, and 74.5 ± 1.89%, respectively. And for RLE-6TN cells treated with 2 μg/mL QDs, the uptake efficiency of the three QDs are 67.1 ± 0.95%, 48.6 ± 2.03%, and 32.6 ± 2.14%, respectively. From the data we can see that for both cells, the uptake efficiency of QDs terminated with hydroxyl is relatively lower than the QDs modified with carboxyl or amino, which is consistent with fluorescence imaging results. Nevertheless, for 20 μg/mL QDs treated cells, the uptake efficiency of the three QDs are comparable (all above 97% for HCC-15 and all above 90% for RLE-6TN). These results indicate that the *in vitro* uptake of the InP/ZnS QDs is concentration dependent.

### Effect of InP/ZnS QDs on Cell Viabilities

**FIGURE 4:**
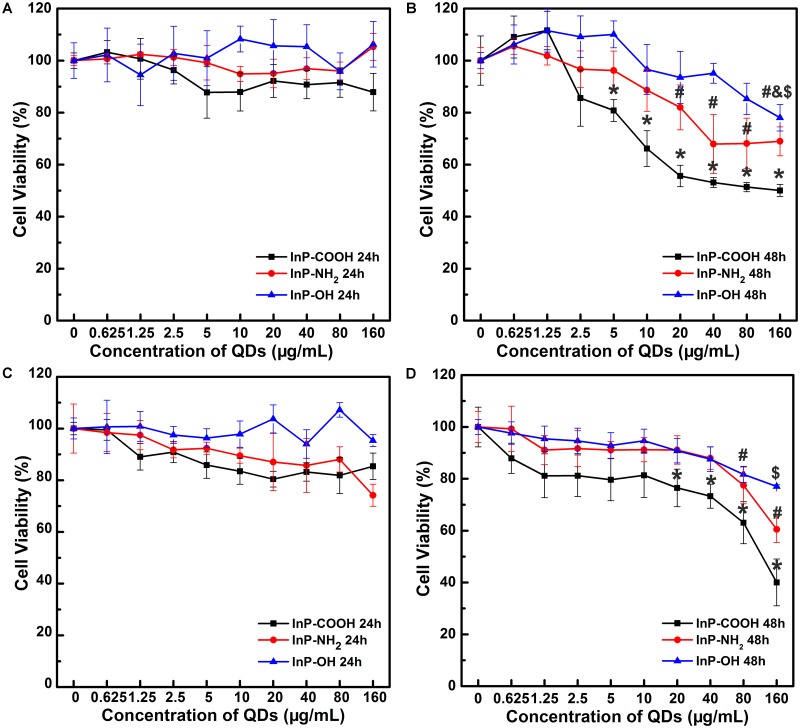
Relative cell viability of HCC-15 **(A,B)** and RLE-6TN cells **(C,D)** treated with (i) InP/ZnS-COOH, (ii) InP/ZnS-NH_2_, and (iii) InP/ZnS-OH QDs, respectively. The cells are incubated with different concentrations of QDs for 24 **(A,C)** and 48 h **(B,D)**. Values are means ± SD, *n* = 6. (^∗^*p* < 0.05 vs. control of InP/ZnS-COOH; #*p* < 0.05 vs. control of InP/ZnS-NH_2_; $*p* < 0.05 vs. control of InP/ZnS-OH).

In order to evaluate the cytotoxicity of the QDs terminated with carboxyl, hydroxyl or amino groups on lung tissue cells, MTT assays were conducted on HCC-15 and RLE-6TN cells with QDs for 24 and 48 h. For the case of HCC-15 cells treated for 24 h, the cell viability of the three groups remained above 90% when the applied QD concentrations range from 0.625 to 160 μg/mL (**Figure 4A**). Similar pattern of cell viability trends are shared for RLE-6TN cells treated for 24 h (**Figure 4C**), the cell viability remained above 90% for InP-NH_2_ and InP/ZnS-OH groups and above 80% for InP/ZnS-COOH groups. The results here show that InP/ZnS QDs terminated with carboxyl, hydroxyl or amino groups have no considerable cytotoxicity within 24 h. However, a different trend was observed when we extend the incubation time to 48 h. The cell viability of 48 h post-treatment decreased evidently as QD concentration increased (**Figures 4B,D**), which indicated that the InP/ZnS QDs showed a concentration dependent cytotoxicity pattern in the cell viability.

### InP/ZnS QDs Promote Cell Apoptosis

**FIGURE 5:**
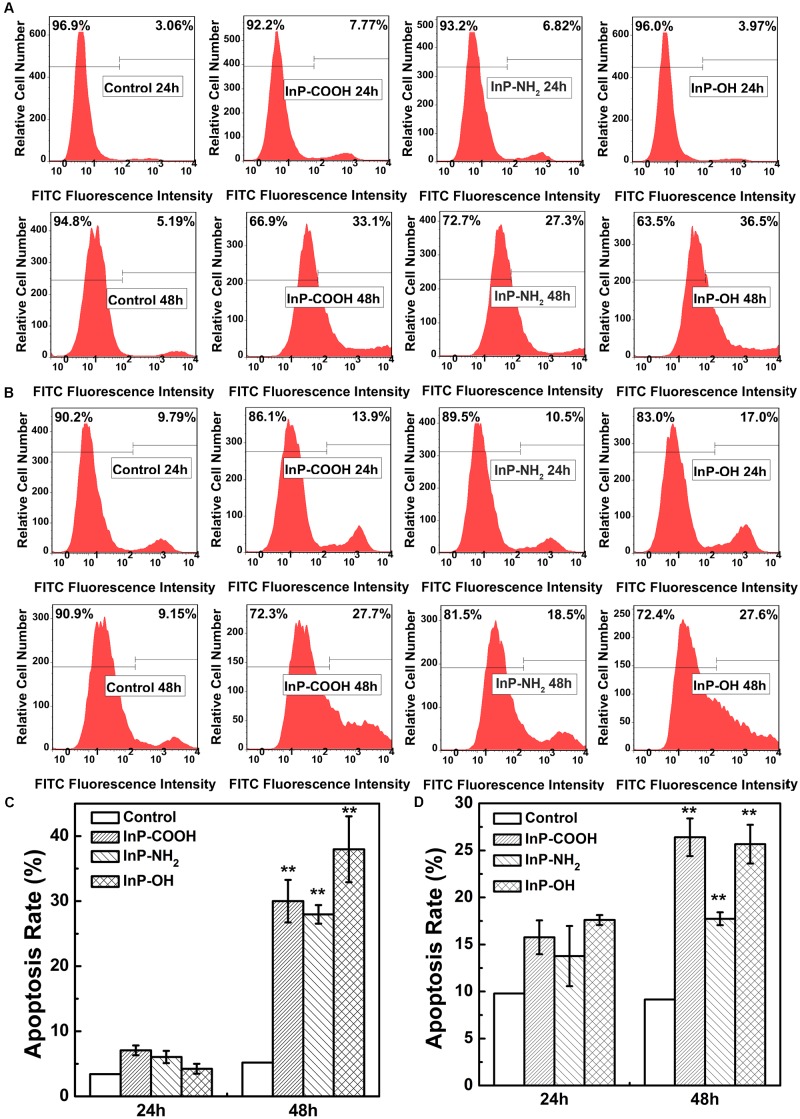
Apoptosis rate of HCC-15 cells **(A,B)** and RLE-6TN cells **(C,D)** treated with (i) Blank, (ii) InP/ZnS-COOH QDs, (iii) InP/ZnS-NH_2_ QDs, and (iv) InP/ZnS-OH QDs respectively. Representative plots of FITC fluorescence intensity in HCC-15 **(A)** and RLE-6TN cells **(C)** treated for 24 and 48 h. The apoptosis rate of HCC-15 cells **(B)** and RLE-6TN cells **(D)**, evaluated from experiments shown in **(A,C)**. Values are means ± SD, *n* = 3. (^∗^*p* < 0.05 vs. control; ^∗∗^*p* < 0.01 vs. control).

To systematically assess the toxicity of InP/ZnS QDs on cells, the fraction of apoptotic cells caused by QDs were determined by flow cytometry analysis. **Figures 5A,B** are the representative plots of flow cytometry analysis in HCC-15 and RLE-6TN cells, respectively. For the case of HCC-15 cells treated with 20 μg/mL QDs for 24 h, the cell apoptosis rates of the three QDs groups presented comparable to the control group (**Figure 5C**). However, when the exposure time extend to 48 h, the cell apoptosis rates increased dramatically to 30.0 ± 3.26%, 27.9 ± 1.42%, and 38.0 ± 5.06%, respectively (**Figure 5C**), which presented significant difference compared with the control group (*P* < 0.01). The same set of experiments was performed using the RLE-6TN cell line (**Figures 5B,D**). When compared to the untreated cells, the apoptosis rates of the three QDs treated cells all exhibited remarkable increase (*P* < 0.01) (**Figure 5D**). These results demonstrated that InP/ZnS QDs increased the occurrence of apoptotic events in cells after 48 h treatment.

### InP/ZnS QDs Induce ROS Generation in Lung-Derived Cells

**FIGURE 6:**
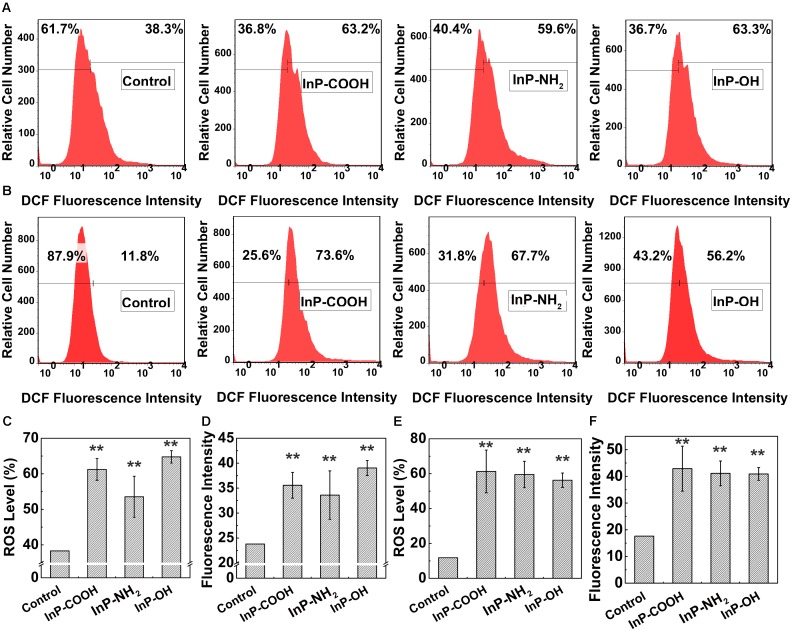
The ROS level in HCC-15 **(A,C,D)** and RLE-6TN cells **(B,E,F)** treated with (i) Blank, (ii) InP/ZnS-COOH QDs, (iii) InP/ZnS-NH_2_ QDs, and (iv) InP/ZnS-OH QDs for 4 h. Representative plots of the DCF fluorescence intensity in HCC-15 **(A)** and RLE-6TN cells **(B)**. The ROS level in HCC-15 **(C)** and RLE-6TN cells **(E)**. The average fluorescence intensity of HCC-15 **(D)** and RLE-6TN cells **(F)**. **(C–F)** are evaluated from experiments shown in **(A,B)**. Values are means ± SD, *n* = 3. (^∗^*p* < 0.05 vs. control; ^∗∗^*p* < 0.01 vs. control).

To further probe the mechanism of cytotoxicity, cellular reactive oxygen species (ROS) levels were measured by flow cytometry analysis. **Figures 6A,B** are the representative plots of flow cytometry analysis in HCC-15 and RLE-6TN cells, respectively. For the case of HCC-15 cells, the fraction of cells generated reactive oxygen were 61.2 ± 3.07%, 53.5 ± 5.78%, and 64.8 ± 1.75%, respectively (**Figure 6C**), which showed significantly difference compared with the control (*P* < 0.01). The average DCF fluorescence intensity was correlated with the amount of ROS generated in the cell. The average fluorescence intensity of the QDs treated cells (35.56 ± 2.58, 33.6 ± 4.86, and 39.0 ± 1.50) were significantly stronger than the untreated cells (*P* < 0.01), which implied plentiful ROS generation in the treated cells (**Figure 6D**). The same set of experiments was performed using the RLE-6TN cell line (**Figures 6E,F**) and the data also presented increased intracellular ROS levels of the experiment groups compared to the control (*P* < 0.01). These results suggested that InP/ZnS QDs had the potential to induce the intracellular ROS generation after being uptake by the cells.

## Discussion

In recent years, people have developed many non-cadmium QDs because of their concerns on the risk of cadmium exposure from cadmium-containing QDs. InP/ZnS QDs as frequently used non-cadmium QDs have emerged as a presumably less hazardous alternative to cadmium-based QDs (Kuo et al., 2017). However, toxicity studies on InP/ZnS QDs have just begun, and little has been known about their toxicological effects. Thus, the present study investigated the cytotoxicity of InP/ZnS QDs toward two lung-derived cell lines, HCC-15 and RLE-6TN cells.

It is well known that surface modification and stabilization of QDs appears to be the most key steps to make them hydrophilic and functionalized. The functional groups on the surface make it feasible to cross-link with desired small molecules or ligand of biomolecules (e.g., peptide, antigen or antibody) by means of conjugating. Among these surface coating groups, carboxyl, hydroxyl and amino groups are most frequently used for such biological functionalization (Mashinchian et al., 2014). In this study, QDs with different surface functional groups (carboxyl, hydroxyl or amino groups) were used for the cytotoxicity evaluation.

The different surface functional group of QDs makes them different in water stability and hydrodynamic size distributions. Bantz et al investigated the agglomeration behavior of two SiO_2_-based nanoparticles with different surface functional groups. They found that the negatively charged silica particles are easier to agglomerate than the positively charged ones (Bantz et al., 2014). Here, our data showed that InP/ZnS coated with hydroxyl group were more likely to aggregate, and the particle size in the water was far greater than the other QDs coated with carboxyl or amino group. It is mainly because that the hydroxyl groups around the particles couldn’t provide sufficient steric hindrance to counterbalance the attractive Van der Waal forces between particles (Wiogo et al., 2011). Apart from the stability and hydrodynamic size distribution, the surface properties also influence the fluorescence intensity of the QDs. Nguyen et al reported that the intense photoluminescence in carbon nanodots is originated from abundant surface functional groups (Vanthan et al., 2016). According to the study of Lu et al. (2000) Mn^2+^-doped ZnS nanoparticles exhibit a 30-fold increase in PL intensity after surface passivation by carboxylic functional groups. In our study, InP/ZnS QDs terminated with carboxyl groups exhibits evidently enhanced fluorescence intensity than QDs terminated with hydroxyl or amino groups. It is because the exchange process of carboxyl groups and other ligands caused damage to the surface of the QDs to a certain degree (Xiao et al., 2015).

Different physiochemical properties of these three QDs influenced how they interact with cells and, which induced different toxicological patterns. In the last decade, confocal fluorescence microscopy has emerged as an ultra-sensitive tool to probe the interaction between NPs and cells. For example, in order to identify the effect of the PEG-capped LSMO MNPs on MCF7 cells, Thorat and colleagues carried out confocal microscopy aided with multiple staining to observe the morphology of the cells as well as the induced apoptosis and necrosis (Thorat et al., 2016a). In our study, confocal microscopy is performed to determine the uptake of InP/ZnS QDs by HCC-15 and RLE-6TN cells and suggest the distribution of QDs in cytoplasm. Similar results were obtained from the confocal imaging of mPEG capped IONPs in MCF7 cells (Thorat et al., 2016b). Here we showed that InP/ZnS-COOH and InP/ZnS-NH_2_ were able to enter the cells more easily than InP/ZnS-OH which was possibly owing to different hydrodynamic size and functional groups on the surface. It is known that the nanoparticle size is one important factor in determining the ability of nanoparticles to enter cells. For example, Huo and his colleagues demonstrated that gold nanoparticles (<6 nm) were able to enter the cell nucleus effectively, whereas larger nanoparticles (10 or 16 nm) only penetrate through the cell membrane, and were found only in the cytoplasm (Huo et al., 2014). Saw et al reported the effect of four sizes of cystine/citric acid-coated confeito-like gold nanoparticles (30, 60, 80, and 100nm) on cellular uptake. They showed that cellular uptake was size dependent with the smallest size of gold nanoparticles (30nm) having the highest cellular internalization in MDA-MB231 breast cancer cells (Saw et al., 2018). Besides nanoparticle size, the cellular uptake of nanoparticles is still influenced by many other factors, including shape, zeta potential, specific surface area, surface charge, catalytic activity, the presence or absence of a shell, and functional groups on the surface (Jayagopal et al., 2007; Ma et al., 2013; Nam et al., 2013; Rivera-Gil et al., 2013). For instance, Zheng et al. studied the uptake of CdSe/ZnS QDs with two commonly reported positive charged (polyethylenimine, cysteamine) and two negative charged (dihydrolipoic acid, glutathione) ligands in human keratinocytes, and they found the selective accumulation of CdSe/ZnS QDs with glutathione in vesicles in the mitochondria matrix (Zheng et al., 2017). Manshian et al showed that intracellular uptake levels of NH_2_-QDs and COOH-QDs were very similar after 24 h exposure, NH_2_-QDs mainly remained in the lysosomes, while COOH-QDs appeared to be continuously internalized and transported by both endosomes and lysosomes (Manshian et al., 2017).

Consequently, the cellular trafficking by QDs influenced their toxicity profiles. The nanoparticles that are more easily to penetrate the cells will possibly result in higher cytotoxicity. In this study, InP/ZnS-COOH and InP/ZnS-NH_2_ showed higher cytotoxicity on two lung-derived cell lines than InP/ZnS-OH. Consistently, Pan et al. have investigated the dependence of the toxicity of gold Nanoparticles on their size in the range from 0.8 to 15 nm. The nanoparticles with 15 nm in size have been found to be 60 times less toxic than 1.4 nm nanoparticles for fibroblasts, epithelial cells, macrophages, and melanoma cells (Pan et al., 2007). The researcher proved that differences in the extent of their cellular uptake resulted in differences in consequent toxicological effects. The continuous flux of CdSe/ZnS QDs terminated with carboxylic acid showed their higher toxicity compared to the NH_2_-QDs, resulting in mitochondrial ROS generation and cytoskeletal remodeling (Manshian et al., 2017). It is worth mentioning that, in this study, the toxicity effect of QDs on the two lung-derived cell lines was different. MTT assay showed that RLE-6TN cell line appeared to be more sensitive to QDs treatment. The cytotoxicity of QDs varies by cell type, which is in line with the previous report (Kim et al., 2015). For example, Mortensen et al manifested that ROS responses induced by QD exposure was correlated with the level of QD uptake and was cell type dependent. Keratinocytes appeared to be at greater risk for QD induced ROS generation than melanocytes after pre-exposing cells to UVB (Mortensen et al., 2015).

One of the common cytotoxicity when living organisms are treated with QDs is apoptosis, where many attempts have been made to explain the mechanisms of apoptosis caused by QDs’ use. Excess generation of ROS will result in oxidative stress that would mediate apoptosis. For example, CdTe QDs have been proposed to induce oxidative stress, which plays a crucial role in CdTe QDs-mediated mitochondrial-dependent apoptosis in HUVECs cells (Yan et al., 2016). Luo et al. (2013) reported that QDs in RAG cells increased intracellular ROS levels and induced autophagy, leading to subsequent apoptosis, which suggest that oxidative stress-induced autophagy is a defense/survival mechanism against the cytotoxicity of QD. Furthermore, the activation of cell death receptors and mitochondria-dependent way could onset apoptosis. Singh et al. (2012) demonstrated that CdS QDs induce apoptotic cell death in LNCaP cells via p53, survivin, Bax/Bcl-2 and caspase pathways by alleviating ROS-mediated oxidative stress. Signal transduction also plays an important role in the regulation of apoptosis. Motohashi and Yamamoto (2004) once reported that Nrf2 controlled the transcription of target genes by binding to the antioxidant response element (ARE) located at the enhancer regions of the genes, giving rise to regulations against xenobiotic and oxidative stresses that could induce cell apoptosis.

There are several physicochemical and molecular mechanisms enabling nanoparticles to cause toxicity toward cells. These include ROS generation, DNA damage and membrane perturbation etc. Oxidative stress is considered to be responsible for toxicity triggered by QDs, as it can induce the intracellular production of ROS. According to existing studies, the majority of nanoparticles have been reported to cause excessive ROS generation in affected cells or organs. Our data showed an increase of intracellular ROS level in HCC-15 and RLE-6TN cells after exposure to InP/ZnS QDs, which aligned to some extent with the previous study retaining cadmium-based QDs in other cell types (Wang X. et al., 2016). Lee et al examined the toxicity effect of carboxylic acid-coated QDs (QD 565 and QD 655) on human keratinocytes. The cell viability of keratinocytes was obviously inhibited by these two types of QDs in a concentration-dependent manner. QD-induced intracellular ROS levels resulted in cell apoptosis via blockade of AKT phosphorylation (Lee et al., 2017). Peynshaert and his colleagues showed that PEGylated QDs were significantly more toxic than MPA-coated QDs due to increased ROS production and lysosomal impairment, which next resulted in autophagy dysfunction and cytotoxicity (Peynshaert et al., 2017).

Reactive oxygen species are chemically reactive species containing oxygen. Oxygen atom has two unpaired electrons in separate orbits in its outer electron shell. This electron structure makes oxygen susceptible to radical formation. The sequential reduction of oxygen through the addition of electrons leads to the formation of a number of ROS including O_2_^⋅-^, H_2_O_2_, ^⋅^OH, HOCl, ONOO^-^, and NO. DCFH-DA probe used in our study was considered to be used for detecting intracellular H_2_O_2_ and oxidative stress. It is cell-permeable and is enzymatically hydrolyzed by intracellular esterase to DCFH which is retained in the cell (Halliwell and Whiteman, 2004). Oxidation of DCFH by H_2_O_2_ results in DCF, a fluorescent product which can be monitored by fluorescence-based techniques. However, it is reported that DCFH does not directly react with H_2_O_2_ to form DCF, it can also be oxidized by other ROS, such as ^⋅^OH, ROO^⋅^, O_2_^⋅-^ (Crow, 1997). Due to the existence of several substances that interfere with the formation of DCF, the probe DCFH-DA, when used in cellular systems, cannot be seen as a specific indicator for H_2_O_2_.

Taken together, our results on cell uptake, cell viability, apoptosis and ROS generation indicated that InP/ZnS QDs can enter these two lung-derived cells with exerting obvious cytotoxicity. InP/ZnS-COOH and InP/ZnS-NH_2_ were able to enter the cells more easily than InP/ZnS-OH, in turn caused more toxic to cells. Although InP/ZnS have regarded as a presumably less hazardous alternative to cadmium-based QDs, appropriate concentration and surface functional groups are needed to be optimized for biological and therapeutic applications in the future.

## Conclusion

In summary, we reported the cytotoxicity of InP/ZnS QDs with different surface groups (NH_2_, COOH, OH) toward two lung-derived cell lines, HCC-15 and RLE-6TN cells. The results showed that InP/ZnS-OH was more likely to aggregate, and the particle size in the water was far greater than the other InP/ZnS-COOH and InP/ZnS-NH_2_. InP/ZnS-COOH and InP/ZnS-NH_2_ were able to enter the cells more easily than InP/ZnS-OH. High doses of all these QDs caused the cell viability to decrease, and InP/ZnS-COOH and InP/ZnS-NH_2_ appeared to be more toxic than InP/ZnS-OH. In addition, InP/ZnS QDs treatment presented increased cell apoptosis and enhanced intracellular ROS levels. These results suggested that appropriate concentration and surface functional groups should be optimized when InP/ZnS QDs are utilized for biological and therapeutic purpose in the future.

## Author Contributions

GL designed the experiments. TC carried out the experiments and wrote the manuscript. LL, JW, YC, and ZY assisted with sample collection. GX, XW, and WJ helped with experiment results analysis.

## Conflict of Interest Statement

The authors declare that the research was conducted in the absence of any commercial or financial relationships that could be construed as a potential conflict of interest.
