# Supplementary figures and images for: Cytotoxicity of InP/ZnS Quantum Dots With Different Surface Functional Groups Toward Two Lung-Derived Cell Lines
Source: Front Pharmacol. 2018 Jul 13;9:763. doi: 10.3389/fphar.2018.00763 (PMC6053512; doi:10.3389/fphar.2018.00763)

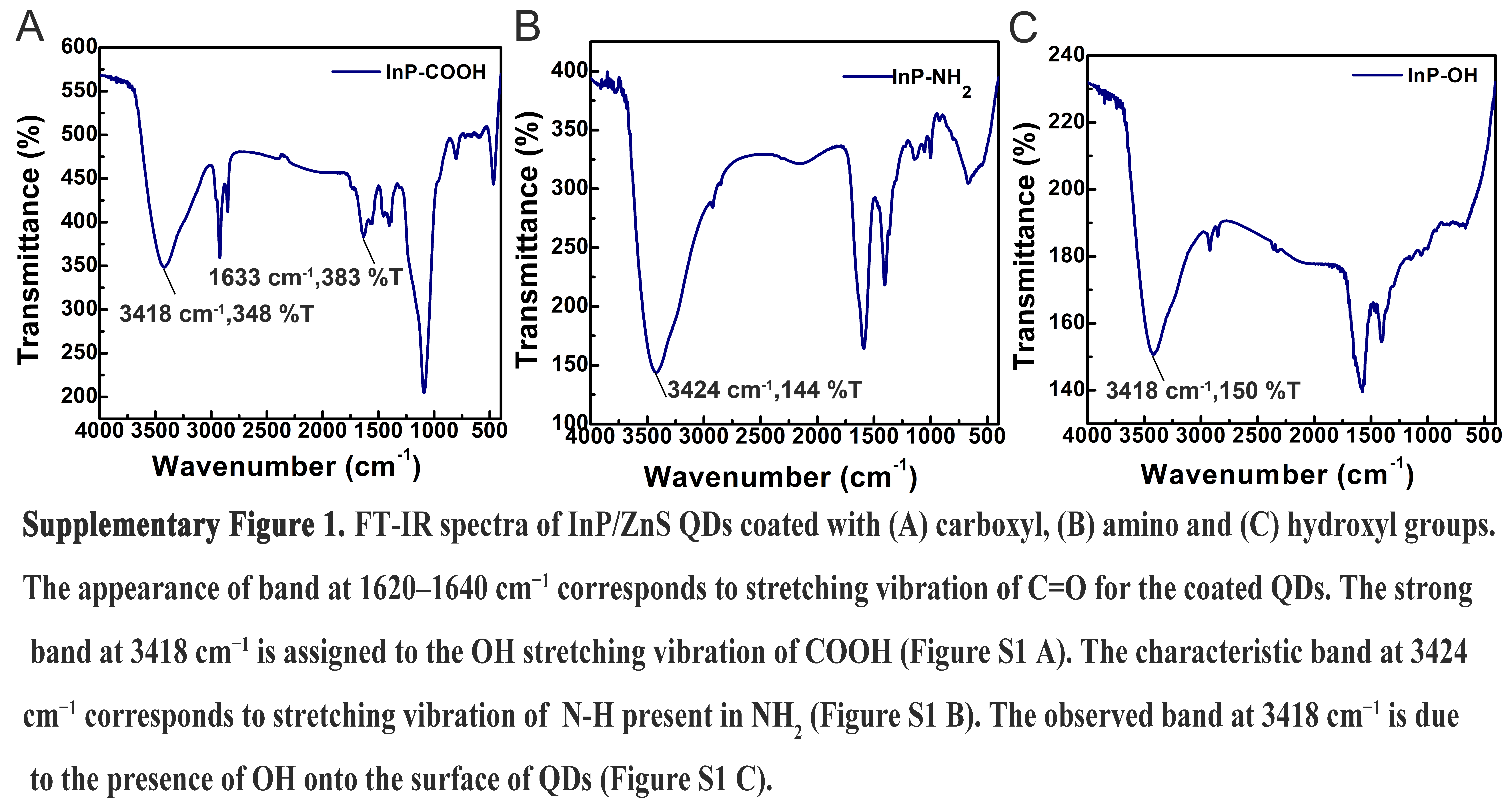

Supplement: Supplementary file 1 [file Image_1.TIF]
